# The strength of selection is consistent across both domains of the MHC class I peptide-binding groove in birds

**DOI:** 10.1186/s12862-021-01812-x

**Published:** 2021-05-08

**Authors:** Piotr Minias, Ke He, Peter O. Dunn

**Affiliations:** 1 Department of Biodiversity Studies and Bioeducation, Faculty of Biology and Environmental Protection, University of Łódź, Banacha 1/3, 90-237 Łódź, Poland; 2 College of Animal Science and Technology, College of Veterinary Medicine, Zhejiang Agriculture and Forestry University, Hangzhou, China; 3 Behavioral and Molecular Ecology Group, Department of Biological Sciences, University of Wisconsin-Milwaukee, Milwaukee, USA

**Keywords:** Birds, Genomics, Major Histocompatibility Complex, MHC, Peptide-binding groove, Selection

## Abstract

**Background:**

The Major Histocompatibility Complex (MHC) codes for the key vertebrate immune receptors responsible for pathogen recognition. Foreign antigens are recognized via their compatibility to hyper-variable region of the peptide-binding groove (PBR), which consists of two separate protein domains. Specifically, the PBR of the MHC class I receptors, which recognize intra-cellular pathogens, has two α domains encoded by exon 2 (α_1_) and exon 3 (α_2_) of the same gene. Most research on avian MHC class I polymorphism has traditionally focused exclusively on exon 3 and comparisons of selection between the two domains have been hampered by the scarcity of molecular data for exon 2. Thus, it is not clear whether the two domains vary in their specificity towards different antigens and whether they are subject to different selective pressure.

**Results:**

Here, we took advantage of rapidly accumulating genomic resources to test for the differences in selection patterns between both MHC class I domains of the peptide-binding groove in birds. For this purpose, we compiled a dataset of MHC class I exon 2 and 3 sequences for 120 avian species from 46 families. Our phylogenetically-robust approach provided strong evidence for highly consistent levels of selection on the α_1_ and α_2_ domains. There were strong correlations in all selection measures (number of positively/negatively selected residues and *dN*/*dS* ratios) between both PBR exons. Similar positive associations were found for the level of amino acid polymorphism across the two domains.

**Conclusions:**

We conclude that the strength of selection and the level of polymorphism are highly consistent between both peptide-binding domains (α_1_ and α_2_) of the avian MHC class I.

**Supplementary Information:**

The online version contains supplementary material available at 10.1186/s12862-021-01812-x.

## Background

The Major Histocompatibility Complex (MHC) genes code for the key immune receptors of the adaptive immune system in vertebrates. Their primary function is to bind antigens of intra- and extra-cellular pathogens (MHC class I and class II, respectively), and initiate an immune response upon their recognition. The MHC gene family constitutes a unique evolutionary system, which is characterized by extraordinary polymorphism. In fact, the MHC is recognized as the most polymorphic region in vertebrate genomes [1], reaching thousands of alleles in some natural populations (for example > 14,000 allelic variants described in humans up to date, [2]). This immense diversity is generated and maintained by pathogen-driven balancing selection, which acts on the MHC through several evolutionary mechanisms [3]. First, overdominant selection (heterozygote advantage) promotes higher fitness of heterozygote over homozygote genotypes, as MHC heterozygotes can recognize more antigens and, thus, should be able to mount an immune response against a broader spectrum of pathogens [4, 5] (but see Wegner et al. [6] for the optimal MHC diversity hypothesis). Second, negative frequency-dependent selection promotes higher fitness of rare genotypes, as pathogens should evolve to avoid MHC variants that are most common in the host populations [7]. Third, fluctuating selection acts through a spatial and temporal variation in the fitness of particular genotypes, which should maintain MHC polymorphism due to pathogen-driven selection pressures varying in space and time [8]. Finally, MHC diversity is also shaped by sexual selection (e.g. MHC-based disassortative mating, [9, 10]), which stabilizes allele frequencies, reduces fluctuations in dominant alleles and protects functional variants against drift [11]. Although the relative importance of these mechanisms and their relative contribution to the maintenance of MHC diversity is difficult to assess [3], their joint effects can be traced as the excess of nonsynonymous over synonymous nucleotide substitutions in the MHC gene pool within populations [12].

MHC receptors bind antigens at the peptide-binding groove, which is expected to be the primary target of balancing selection within the MHC. Peptide-binding regions (PBR) are formed by two molecular domains: α_1_ and α_2_ in MHC class I (coded by exon 2 and exon 3 of the same MHC class I gene) [13, 14] or α_1_ and β_1_ in MHC class II (coded by exon 2 of MHC class II genes) [15]. Since it is difficult or impossible to sequence both PBR exons in one sequencing run (at least with traditional Sanger methodology), a standard approach in MHC research on non-model species is to target only one of the PBR exons. Studies on the MHC class I in birds have typically focused on exon 3 (e.g. [16–19]), while almost no information exists on the polymorphism of exon 2 in natural populations of birds [20]. The choice of exon 3, although subjective, has been enhanced by a rapid development of conservative degenerate and taxa-specific primers, allowing amplification of this part of the MHC class I sequence in a broad spectrum of avian lineages [21–23]. However, to the best of our knowledge it is not known if estimates of polymorphism and selection inferred from exon 3 are representative for the entire MHC class I PBR in birds. Addressing this hypothesis has long been hampered by the scarcity of available MHC class I exon 2 sequences, but the unprecedented development of genomic resources in the recent years allows us to retrieve extensive data on both MHC class I PBR domains for a wide spectrum of non-model species. Here, we used available genomic data to compile MHC class I exon 2 and exon 3 sequences for 120 avian species from ca. 60% extant orders and tested for the differences in selection patterns and amino acid polymorphism between both MHC class I PBR domains.

## Results

**Fig. 1:**
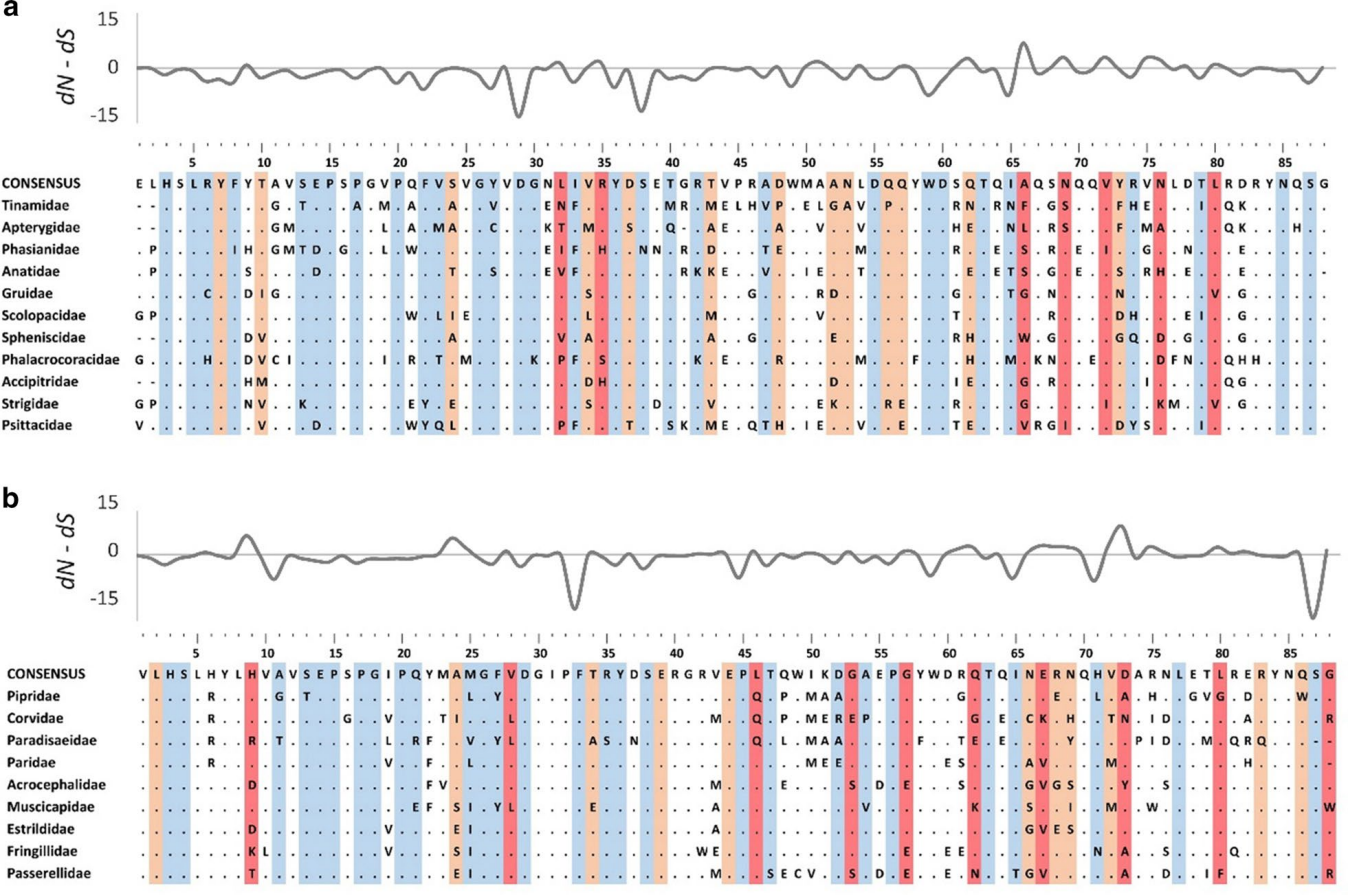
Alignments of amino acid sequences of MHC class I exon 2 in non-passerine (**a**) and passerine (**b**) birds. Dots indicate amino acids identical with the reference consensus sequence. Residues under pervasive positive selection (FUBAR/FEL) are marked with dark red, residues under episodic positive selection (MEME) and marked with light red, while residues under negative selection (FUBAR/FEL) are marked with blue. Variation in selection parameter (*dN*–*dS*; FUBAR analysis) is shown above the alignments

**Fig. 2:**
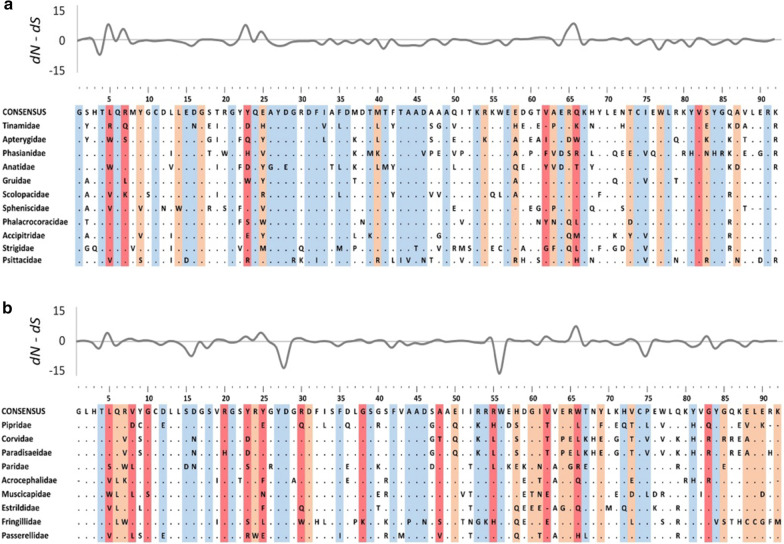
Alignments of amino acid sequences of MHC class I exon 3 in non-passerine (**a**) and passerine (**b**) birds. Dots indicate amino acids identical with the reference consensus sequence. Residues under pervasive positive selection (FUBAR/FEL) are marked with dark red, residues under episodic positive selection (MEME) and marked with light red, while residues under negative selection (FUBAR/FEL) are marked with blue. Variation in selection parameter (*dN*–*dS*; FUBAR analysis) is shown above the alignments

**Fig. 3:**
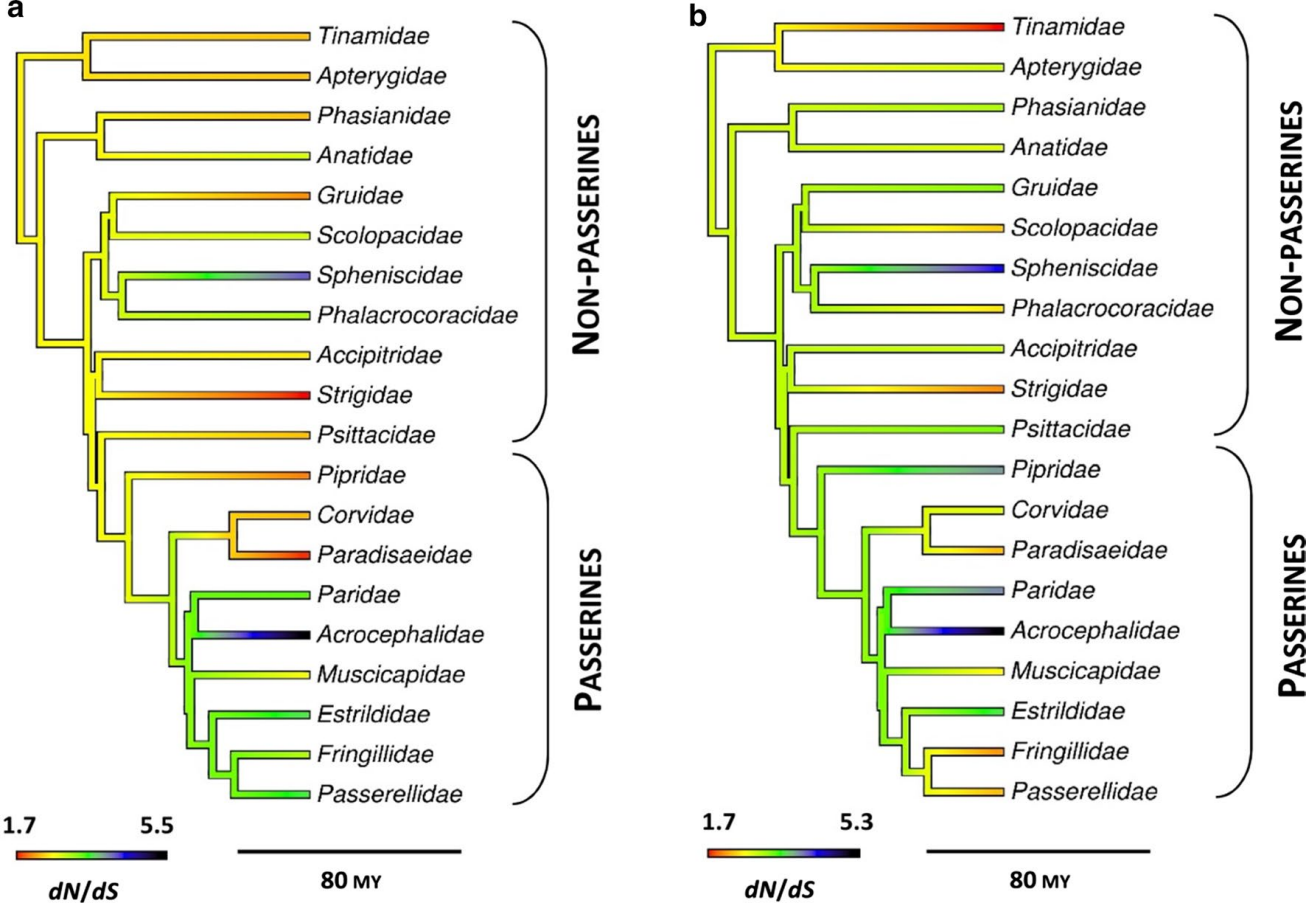
Selection signature (*dN*/*dS* ratio) at the MHC class I exon 2 (**a**) and exon 3 (**b**) across 20 avian families

Selection across the entire dataset was relatively consistent between MHC class I exon 2 and exon 3. Although non-synonymous vs. synonymous nucleotide substitution rates (*dN*/*dS* ratio) were higher for exon 3 than exon 2 (2.90 vs. 2.05, Table 1), there was a slightly higher number of positively selected residues at exon 2 (Table 1). At both exons, passerines had a stronger signature of diversifying selection (number of positively selected residues and *dN*/*dS* ratios) than non-passerines (Figs. 1, 2 and 3). Positions of positively selected residues were significantly repeatable between both lineages at exon 3 (R = 0.37, 95% CI 0.18–0.53, P < 0.001), but not exon 2 (R = 0.03, 95% CI − 0.18–0.24, P = 0.402) (Figs. 1, 2). Positions of negatively selected residues were significantly repeatable at both exons (R = 0.30, 95% CI 0.10–0.48, P = 0.002 for exon 2; R = 0.40, 95% CI 0.21–0.55, P < 0.001). The strongest signature of diversifying selection at both exon 2 and 3 (as measured with *dN*/*dS* > 4) was found in Acrocephalidae and Spheniscidae (Fig. 3). These families also had some of the highest numbers of positively selected residues identified at both exons (≥ 6 per family per exon). The weakest signature of diversifying selection (*dN*/*dS* < 2; ≤ 2 positively selected residues) was recorded in Paradisaeidae and Strigidae (exon 2) or Tinamidae (exon 3) (Fig. 3). The signature of selection was not affected by genome assembly quality, as indicated by very high repeatability of codon-specific *dN* and *dS* estimates between the datasets with and without sequences retrieved from low quality genomes (0.93 < R < 0.99, all P < 0.001).

**Table 1:** Selection signature at the MHC class I exon 2 and exon 3 in passerine and non-passerine birds

Exon	Length (bp)	Lineage	No. species (families)	No. residues			*dN*/*dS* ratio	
				Pervasive positive selection (FUBAR/FEL)	Episodic positive selection (MEME)	Negative selection (FUBAR/FEL)	All residues	Positively selected residues
2	264	Non-passerines	63 (28)	6/7	20 (13)	32/32	0.54	2.04
		Passerines	57 (18)	9/10	20 (11)	26/25	0.64	2.83
		All	120 (46)	11/12	27 (16)	37/37	0.54	2.05
3	276	Non-passerines	63 (28)	4/6	19 (13)	37/39	0.67	2.59
		Passerines	57 (18)	12/13	30 (17)	25/25	0.64	2.97
		All	120 (46)	7/10	32 (22)	35/38	0.74	2.90

The numbers of residues under pervasive positive and negative selection were inferred with FUBAR and FEL approaches, while the number of residues under episodic positive selection was inferred with MEME approach. The number of residues under episodic positive selection (MEME) that were not recognized as under pervasive positive selection (FUBAR or FEL) was given in parentheses. Nucleotide substitution rates (*dN*/*dS* ratio) were inferred for all residues and for 20 most positively selected residues

**Fig. 4:**
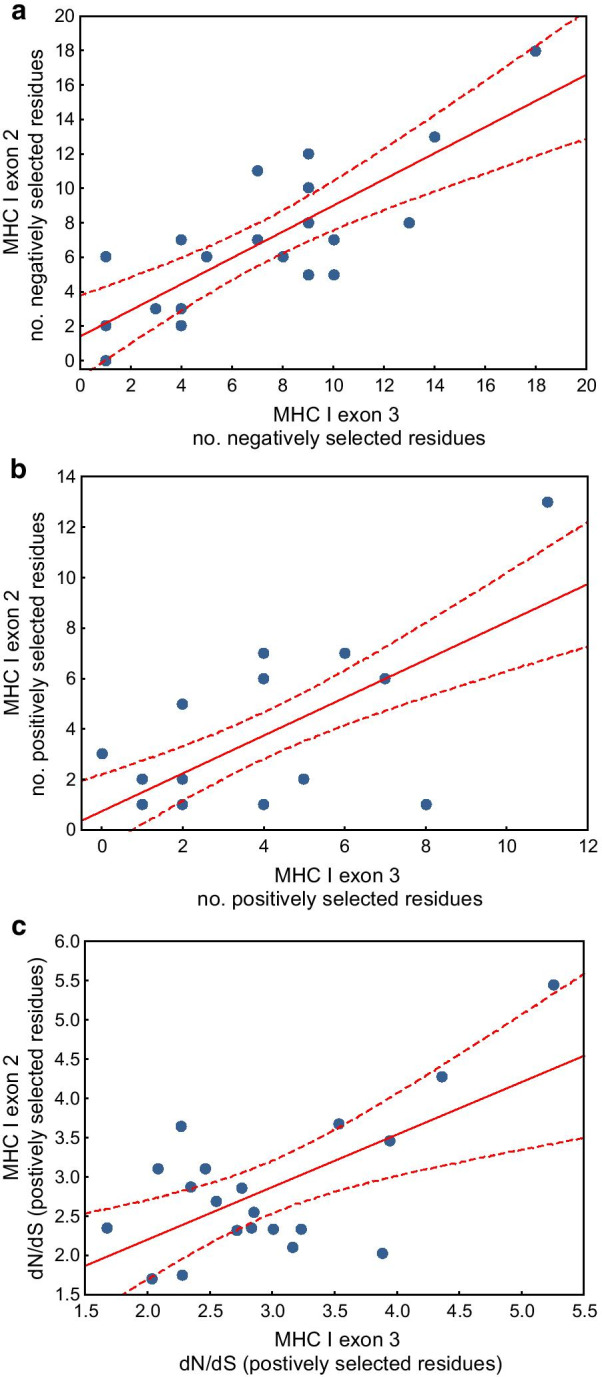
Linear associations in selection signature (**a** the number of negatively selected residues, **b** number of positively selected residues, **c** *dN*/*dS* ratio for 20 most positively selected residues) between MHC class I exon 2 and exon 3 across 20 avian families. Linear regressions (solid lines) with 95% confidence intervals (dashed lines) are shown

**Fig. 5:**
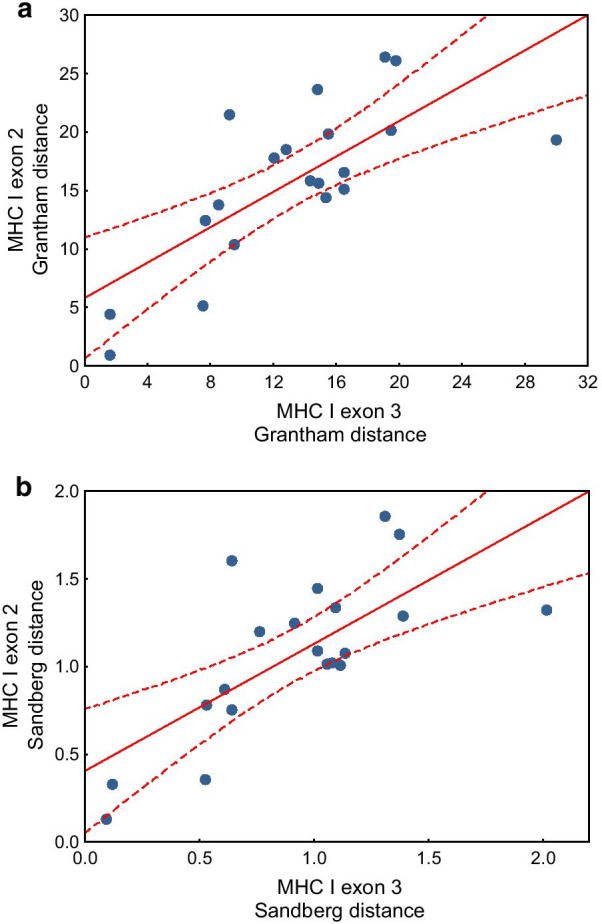
Linear associations between amino acid polymorphism (**a** Grantham distance; **b** Sandberg distance) at MHC class I exon 2 and exon 3 across 20 avian families. Linear regressions (solid lines) with 95% confidence intervals (dashed lines) are shown

Using the phylogenetically-informed approach, we found significant positive linear associations between selection signature at MHC class I exon 2 and exon 3 across 20 avian families. These associations were highly significant for all three measures of selection, including the number of negatively selected residues (β = 0.68, 95% CI 0.42–0.92, P < 0.001; Fig. 4a), number of positively selected residues (β = 0.70, 95% CI 0.28–1.13, P = 0.001; Fig. 4b), and *dN*/*dS* ratios (β = 0.67, 95% CI 0.26–1.08, P = 0.002; Fig. 4c) (Additional file 1: Table S1). At the same time, we recorded no significant differences in the signature of selection at the family level between exon 2 and exon 3 (Additional file 1: Table S2). Positive linear associations at the family level were also found between amino acid polymorphism of exon 2 and 3, as measured with Grantham and Sandberg amino acid distances (Grantham distance: β = 0.77, 95% CI 0.42–1.12, P < 0.001; Sandberg distance: β = 0.74, 95% CI 0.39–1.10, P < 0.001; Fig. 5) (Additional file 1: Table S1). While no significant differences were found between the two exons in Sandberg distance, lower mean Grantham distance was detected at exon 3 when compared with exon 2 (β = − 2.56, 95% CI − 5.03 to − 0.10, P = 0.041) (Additional file 1: Table S2).

## Discussion

The results of our comparative analysis of nucleotide substitution rates at MHC class I exon 2 and exon 3 in birds indicated that selection patterns are highly consistent across both α domains of the peptide-binding groove. Most importantly, we found strong positive associations in all quantified selection measures, i.e. the number of positively/negatively selected residues and *dN*/*dS* ratios, between the two domains across 20 avian families. Similarly, we found positive associations between the level of amino acid polymorphism at the two exons.

So far, analyses of selection and polymorphism patterns at the MHC class I exon 2 in birds were scarce and taxa-specific. One notable exception is a study of the red-billed gull *Larus scopulinus*, in which the entire exon 2 and 3 (ca. 90 residues per exon) region was targeted and compared in a locus-specific approach [20]. The overall strength of positive (diversifying) selection was similar between exon 2 and 3 at the most polymorphic locus (12 positively selected residues per exon), while at the remaining three less polymorphic loci there was a weaker signal of diversifying selection at exon 2 than exon 3 (0–1 vs. 4–7 positively selected residues, respectively) [20]. This seems to suggest that selection acted consistently across both PBR domains at the major classical MHC class I locus in the red-billed gull, while this pattern was disrupted in the minor classical or non-classical loci that were the secondary targets of pathogen-driven selection. Genotyping of MHC class I exon 2 and 3 in another Charadriiform species, the red knot *Calidris canutus*, also provided support for consistency in selection between both PBR domains (six and seven positively selected residues at exon 2 and 3, respectively) [24]. It is, however, worth noting that nucleotide substitutions rates were not directly compared between both exons in either of these two studies. Comparisons of *dN*/*dS* ratios between PBR domains are available for at least two other non-passerine species, yielding contrasting results. Nucleotide substitution rates were relatively similar across both exons (slightly higher *dN*/*dS* at exon 3) in the Humboldt penguin *Spheniscus humboldti* [25], but the excess of nonsynonymous mutations was much more apparent at exon 2 than 3 across the two genotyped MHC class I loci in the golden pheasant *Chrysolophus pictus* [26]. Although the last example indicates that selection may impact α_1_ and α_2_ domain differently in some taxa, most taxa-specific studies seem to support our conclusions that the strength of selection is generally similar at both MHC class I PBR domains in birds. Consistent with selection patterns, we found strong positive associations in amino acid polymorphism between the two exons, indicating that similar patterns of diversity are maintained at both PBR domains. At the same time, mean Grantham distance was slightly higher at exon 2 than exon 3, providing no evidence for depleted polymorphism at MHC class I α_1_ domain.

Although our comparative analysis provided support for general consistency in selection patterns across both MHC class I PBR domains, it needs to be acknowledged that both exons may vary in their ability to recognize specific antigens and, thus, confer resistance to different pathogens and parasites. For example, exon 3 was suggested to play a more important role than exon 2 in resistance to Marek’s disease virus (MDV) in the domestic chicken *Gallus gallus* [27]. Chickens infected with MDV had much lower *dN*/*dS* ratio at exon 3 when compared with uninfected individuals, whereas the difference in nucleotide substitution rate at exon 2 between both groups of chickens was much less apparent [27]. In contrast, polymorphisms at both exon 2 and 3 were associated with primary antibody response of chickens to *Salmonella enteritidis* and *Brucella abortus* [28]. Research linking MHC class I polymorphism with disease resistance in wild birds has focused exclusively on exon 3, providing strong empirical support for associations between genetic variation at this exon and resistance to avian malaria. MHC class I exon 3 alleles or supertypes have been reported to confer qualitative and quantitative resistance to various *Plasmodium* and *Haemoproteus* strains in a wide spectrum of wild passerine species, including reed warblers [29, 30], sparrows [31–33], and tits [34, 35]. To the best of our knowledge, similar studies for MHC class I exon 2 are lacking and we recommend that they should be empirically tested in the future research on the avian MHC.

In conclusion, our analysis based on genomic resources showed that nucleotide substitution rates and amino acid polymorphism measures are well correlated between the two MHC class I PBR domains and, thus, the strength of selection acting at exon 3 should be roughly representative for selection at the entire PBR. We believe this constitutes an important methodological consideration in avian MHC research, as most studies on MHC class I in non-model bird species have focused on a single (α_2_) PBR domain coded by this exon. At the same time, it should be kept in mind that our results are of purely correlative nature and we also acknowledge that some variation in selection may possibly occur between exon 2 and 3 at the species-specific level. Also, despite the fact that pathogen-driven balancing selection may act with similar strength on both MHC class I PBR domains, each domain can have a different role in recognition of different antigens. Consequently, exon 2 and 3 may show a different functional importance in immune response against specific pathogens. Thus, while our results validated the traditional approach to estimate the strength of selection at MHC class I in wild birds, we recommend that re-focusing research efforts from a single PBR domain to both PBR domains could possibly provide novel insights into the functional variation and evolutionary trajectories of the avian MHC. An increasing use of long-read sequencing may help facilitate these analyses.

## Methods

### Data compilation

**Fig. 6:**
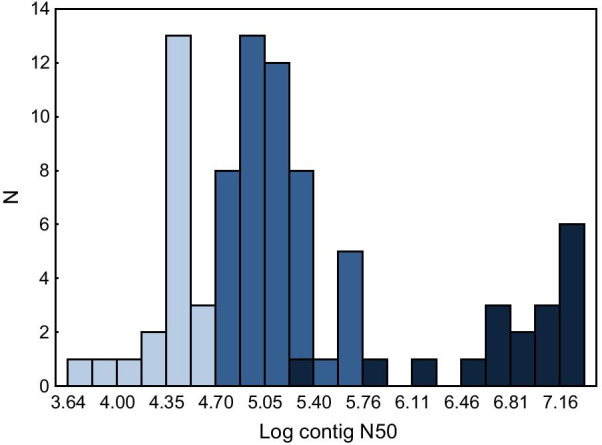
Assembly quality (contig N50) of avian genomes used to retrieve MHC class I exon 2 and 3 sequences. Light blue—25% low quality genome assemblies, dark blue—high quality NGS genome assemblies, navy blue—TGS genome assemblies

To assess selection across both domains of the peptide-biding region of MHC class I in birds, we compiled a dataset of exon 2 and exon 3 sequences deposited in publicly available databases of the National Center for Biotechnology Information (NCBI, Bathesda, MD, USA). We primarily used the Genome NCBI database to retrieve the sequences, because there are relatively few sequences of exon 2 from targeted studies of the MHC deposited in GenBank (198 sequences for exon 2 versus 6894 sequences for exon 3; accessed on 22.11.2020). First, we used a database of exon 2 and 3 sequences retrieved from genomes assembled based on next- and third-generation sequencing (NGS and TGS) data, which was previously used to examine MHC copy number variation in birds [36]. Briefly, we used consensus avian MHC class I sequences generated from family- and order-level alignments to Blast-search exons 2–4 in the available genomic resources (details in He et al. [36]). In total, we retrieved MHC class I exon 2 and 3 sequences of 67 and 18 species from NGS and TGS genomes, respectively. The mean (± SE) contig N50 of NGS and TGS genomes was 0.12 ± 0.01 Mb and 10.3 ± 1.6 Mb, respectively (Fig. 6), while scaffold N50 was 8.8 ± 1.9 Mb (NGS genomes) and 44.5 ± 7.3 Mb (TGS genomes). The mean genome coverage was similar between both methods (88.8 ± 6.8 vs. 79.9 ± 8.4 for NGS and TGS, respectively; t-test: P = 0.52). In total, we retrieved 204 exon 2 and 167 exon 3 sequences from genomic resources and they originated from 161 and 131 different scaffolds, respectively. The mean length of scaffolds used to retrieve data did not differ between exon 2 and 3 (383.1 ± 99.2 kb vs. 353.1 ± 107.2 kb, respectively; t-test: P = 0.84). Genomic data were complemented with sequences of another 35 species retrieved from the Nucleotide database at NCBI. To avoid unbalanced sample sizes, we limited the number of sequences to 10 per species and compiled the same number of sequences for each exon (n = 330). The final database had a wide phylogenetic coverage and was represented by 120 species from 46 families and 22 orders (ca. 25% of all extant families and ca. 60% of all extant orders; [37]). On average, there were 7.16 ± 0.87 [SE] sequences and 2.61 ± 0.47 [SE] species available per family.

Previous analyses of selection at the avian MHC revealed contrasting selection patterns between two major avian lineages, passerines and non-passerines [38], resulting from different evolutionary trajectories of the MHC (e.g. higher duplication rate in passerines [39]). Thus, we performed selection analyses both across all species and separately for passerine and non-passerine birds. Both lineages had similar sample sizes in terms of species numbers (57 vs. 63 for passerines and non-passerines, respectively) and sequence numbers (114 vs. 126 for passerines and non-passerines, respectively), which minimized biases in selection inference resulting from unbalanced samples. To check for the effect of genome assembly quality on our results, we have re-run the analyses (across all species) using a subsample of sequences retrieved from 75% of genomes with the highest contig N50 values (> 0.05 Mb) (Fig. 6). To quantify repeatability of the results, we calculated intra-class correlation coefficients for selection signal (codon-specific *dN* and *dS* estimates, see below) between the two datasets using the *irr* package [40] developed for R statistical environment (R Foundation for Statistical Computing, Vienna, Austria).

In a separate analysis, we examined differences in selection between exon 2 and exon 3 at the family level. Here, we extracted separate datasets for 20 families, in which at least four sequences per exon from at least two species were available (per family). In one case, we combined sequences of two sister Psittaciformes families (Psittacidae and Strigopidae) to meet these thresholds. The average sample size for this analysis was 4.60 ± 0.91 [SE] species and 13.03 ± 2.22 [SE] sequences per family. We did not conduct analyses at the species level, because the sample sizes were small at the within-specific level (on average 2.75 ± 0.14 [SE] sequences per species, > 50% species with 1–2 sequences) and sequence variants sampled within the same population may represent segregating polymorphisms, leading to biases in selection inference [41].

### Recombination signal

Recombination (including gene conversion) is an important molecular mechanism generating allelic variation at the MHC, because it creates new haplotypes by shuffling existing variation within and between loci [42]. Since recombination may affect tree topologies used to infer nucleotide substitution rates [43], we identified recombinant sequences within our dataset prior to selection inference. For this purpose, we used seven different algorithms: Maxchi [44], BootScan [45], Genconv [46], SiScan [47], RDP [48], Chimaera [49], and 3Seq [50], all implemented in RDP v.4.97 software [51]. Recombination analyses were run using default settings and statistical significance threshold of P = 0.05 with Bonferroni correction for multiple comparisons. Recombinant sequences were recognized under a conservative approach, i.e. when recombination signal was supported by at least three of seven algorithms. Since recombination signal may be retained beyond species divergence, we conducted analyses for the entire dataset, separately for exon 2 and exon 3. No recombinant exon 3 sequences were detected within our data, while the analysis of exon 2 data revealed the presence of three different recombination events and nine recombinant sequences within the orders of Anseriformes, Galliformes, and Passeriformes. All these sequences were discarded prior to selection analyses.

### Selection inference

Signature of selection was inferred based on the nonsynonymous versus synonymous nucleotide substitution rates (*dN*/*dS*). In general, nonsynonymous mutations accumulate at a faster rate under positive (diversifying) selection (*dN*/*dS* > 1), while they are expected to be removed and accumulate at a slower rate under negative (purifying) selection (*dN*/*dS* < 1). Similar rates of accumulation of nonsynonymous and synonymous mutations (*dN*/*dS* = 1) indicate no signature of selection under neutral evolution. Nucleotide substitution rates were quantified using a codon-specific approach. Pervasive (constant across the entire tree topology) diversifying and purifying selection was assessed with both Bayesian and maximum-likelihood (ML) algorithms, Fast Unconstrained Bayesian AppRoximation (FUBAR) [52] and Fixed Effect Likelihood (FEL) [53], while episodic (detectable at a proportion of tree branches) diversifying selection was assessed exclusively with the ML algorithm, Mixed Effects Model of Evolution (MEME) [54]. All analyses were run with default settings via the Datamonkey web server [55]. All input trees were inferred from sequence alignments. The analyses were conducted across all data and at the level of two major avian lineages (passerines vs. non-passerines), where residues with posterior probabilities > 0.95 (FUBAR) or statistical significance P < 0.05 (FEL, MEME) were considered to have enough support for selection signal. Positively and negatively selected residues were recognized when identified with at least one of the algorithms. To infer selection at the family-level we used only FUBAR, which is robust against model misspecifications and leaves the distribution of selection parameters essentially unconstrained [52]. Here, because of relatively small sample sizes we adopted a less conservative approach to identify residues under selection (posterior probabilities > 0.90). We used the number of positively selected residues, the number of negatively selected residues and *dN*/*dS* ratios calculated across the 20 most positively selected residues as the measures of selection signal.

### Amino acid polymorphism

To quantify amino acid polymorphism of exon 2 and 3 at the family level we calculated Grantham and Sandberg distances [56, 57] between all available sequences within each family. We used these measures of polymorphism instead of nucleotide diversity, as they take physio-chemical proprieties of amino acids into account. Pairwise distances were computed separately for each exon using *DistCalc* function from the *MHCtools* R package [58] and, then, averaged within each family.

### Statistical analyses

We used a phylogenetically-informed comparative approach to compare selection between exon 2 and 3 across 20 avian families, as different phylogenetic lineages (families) may share evolutionary history to a varying degree and, thus, cannot be treated as statistically independent units. For this purpose, we used Bayesian phylogenetic mixed models [59], as implemented in the *MCMCglmm* R package [60]. First, we tested for linear associations between selection and amino acid polymorphism of exon 2 and exon 3 across all families. Each of selection measures (number of negatively selected residues, number of positively selected residues, *dN*/*dS* ratio) and amino acid polymorphism measures (Grantham and Sandberg amino acid distances) for exon 2 was entered as a response variable in a separate MCMCglmm model, while the same measure for exon 3 was entered as a covariate. Second, we tested for the differences in selection and amino acid polymorphism between the exons, where each measure of selection/polymorphism was entered as a response variable in a separate model (data for both exon 2 and 3), while exon identity was entered as a two-level fixed factor. To control for any possible biases in the sampling effort between the families, the number of sequences was entered as a covariate, while to control for phylogeny, the effect of family was entered as a random factor in all MCMCglmm models. Phylogenetic relationships between families were reconstructed based on the complete avian time-calibrated phylogeny [61] and a backbone tree developed by Ericson et al. [62], as available at the BirdTree web server (http://www.birdtree.org). To account for phylogenetic uncertainty, each model was run for 100 alternative trees and the results were summarized in the *mulTree* R package [63]. Uninformative priors (variance set to 1 and belief parameter set to 0.002) were used for both fixed and random effects. Two chains with 200,000 iterations were run in each analysis. Burn-in period was set to 50,000, and thinning value was set to 100, yielding 1500 samples per model. The two independent chains converged each time, as assessed with potential scale reduction values < 1.1 [64]. Statistical significance of each predictor was inferred with z-score (estimate/SE) test.

## Supplementary Information

**Additional file 1: Table S1.** The results of Bayesian phylogenetic mixed models (MCMCglmm) testing linear associations between selection signal and functional polymorphism of MHC class I exon 2 and exon 3 across 20 avian families. Phylogenetic and residual variance estimates are reported for each model. Estimates with 95% credible intervals, z score values, and P values are presented for each predictor; significant predictors are marked in bold. **Table S2.** The results of Bayesian phylogenetic mixed models (MCMCglmm) testing differences in selection signal and functional polymorphism between MHC class I exon 2 and exon 3 across 20 avian families. Phylogenetic and residual variance estimates are reported for each model. Estimates with 95% credible intervals, z score values, and P values are presented for each predictor; significant predictors are marked in bold.

**Additional file 2. **Sequence list.

## Data Availability

No original data were generated in this study. List of publicly available sequences used in this study is attached as Additional file 2.

## Abbreviations

FEL: Fixed Effect Likelihood
FUBAR: Fast Unconstrained Bayesian AppRoximation
MDV: Marek’s disease virus
MEME: Mixed Effects Model of Evolution
MHC: The Major Histocompatibility Complex
ML: Maximum-likelihood
NCBI: National Center for Biotechnology Information
NGS: Next-generation sequencing
PBR: Peptide-binding region
TGS: Third-generation sequencing
